# A Cross-Sectional Study of Postgraduate Orthodontic Students’ Moral Reasoning Ability and Opinions on Professionalism and Dental Board of Australia Tribunal Outcomes

**DOI:** 10.3390/dj14050307

**Published:** 2026-05-18

**Authors:** Maurice J. Meade, Xiangqun Ju, David Hunter, Lisa Jamieson

**Affiliations:** 1 Orthodontic Unit, School of Dentistry, College of Health, Adelaide University, Adelaide, SA 5000, Australia; 2 Australian Research Centre for Population Oral Health, School of Dentistry, College of Health, Adelaide University, Adelaide, SA 5005, Australia; xiangqun.ju@adelaide.edu.au (X.J.); lisa.jamieson@adelaide.edu.au (L.J.); 3 Office of Health, College of Health, Adelaide University, Adelaide, SA 5000, Australia; david.hunter@adelaide.edu.au

**Keywords:** dental students, dentistry, moral reasoning, orthodontics, postgraduate dental education, professionalism

## Abstract

**Background/Objectives**: The aim of this investigation was to determine postgraduate orthodontic students’ moral reasoning ability and evaluate their opinions on professionalism and the Dental Board of Australia’s (DBA) tribunal outcomes. **Methods**: Students undergoing postgraduate orthodontic specialist training in five Australian universities were invited to participate in a three-part electronic questionnaire survey. Part 1 related to participant demographic details. Part 2 invited responses to a series of statements related to professionalism and 10 DBA tribunal outcomes, and Part 3 was a validated moral reasoning assessment instrument [Defining Issues Test (DIT-2)]. **Results**: A response rate of 80.4% (n = 37) was recorded. The mean DIT-2 Post-conventional Schema (P) score was 31.14 (SD = 13.25). All respondents (n = 37; 100%) were aware of the DBA’s Code of Conduct (Code). Respondents were broadly supportive of the statements related to professionalism although 15.4% reported that being bound by the Code meant they could not fully value the student experience whilst at university. Most respondents considered that most DBA tribunal outcomes were fair or tended towards being lenient. However, one outcome regarding dishonest evidence by a dentist in court related to an employment dispute was considered harsh or very harsh by 53% (n = 20). There was little correlation between P scores and responses to most professionalism statements/DBA outcomes. **Conclusions**: The moral reasoning scores were lower than what might be expected from individuals in the provision of healthcare. The introduction of formal training in moral reasoning may develop postgraduate moral reasoning skills and DIT-2 scores.

## 1. Introduction

Dental practitioners registered with the Australian Health Practitioner Regulation Agency (AHPRA) have a duty to be familiar with and apply the Code of Conduct (Code), which is overseen by the Dental Board of Australia (DBA) [1]. Adherence to the Code ensures that the core regulatory objective, the protection of the public, is maintained [2]. Principle Eight of the Code requires the registrant to demonstrate a standard of professional behaviour that upholds the respect and trust of society, including acting in an honest and ethical manner [3]. A concern regarding a practitioner’s ability to practise safely can be made to AHPRA. This is called a notification [2]. If the concern relates to professional misconduct or the improper obtainment of practitioner registration, the DBA can refer the concern to a tribunal for a hearing. This is analogous to the UK’s ‘fitness to practise’ process overseen by that country’s health professional regulatory authority, the General Dental Council (GDC) [4]. There are several potential outcomes from a tribunal hearing, including suspension to cancellation of the practitioner’s registration [5].

However, while there may be a broad understanding of what is meant by professionalism and ethical conduct, clarity is lacking regarding the concepts of professionalism and ethics as they relate to healthcare [4,6,7]. Shaw determined that being professional means satisfying the standards of the profession of which one is a part [4]. In an Australian dental context, that means meeting the standards of the DBA’s Code. If the standards are breached to such an extent that the registrant is not deemed a ‘fit and proper person’ in a regulatory sense, then the individual is prohibited from practising [8]. Behaving ethically, however, corresponds with being professional *and* being able to contend with morally challenging issues [4]. Although a definition of ‘fit and proper person’ is not provided in the National Law, case law has determined that the expression largely relates to ‘moral integrity’ and it is in this context that it is employed in disciplinary processes in DBA tribunals [8,9].

Moral reasoning employs critical evaluation to specific circumstances to decide what is right or wrong, and what a person ought to do [10]. It is one of the four psychological capacities that underpin the theory of moral behaviour developed by Rest and colleagues from Kohlberg’s stages of moral development [11,12]. The other capacities are moral sensitivity, moral implementation, and moral motivation [10]. Moral reasoning skills are necessary when clinicians process contradictory personal opinions, the wishes of patients and their families and external factors, such as availability of funding and resources, to inform their behaviours [13].

Although it is just one aspect of moral behaviour, it is of crucial significance [14]. Moral reasoning has been categorised into three schemas [11]. The schemas represent overlapping moral reasoning approaches. All approaches are employed by individuals to some extent when they are reasoning through moral dilemmas. The first schema relates to personal interest (PI) strategies where individuals are motivated principally by their own self-interest and are not concerned about the implications to society at large. The second schema, maintaining norms (MN), refers to strategies that concentrate on maintaining social order and deferring to traditional hierarchal systems. Post-conventional (P) strategies relate to the idea that shared moral ideals inform moral obligations and that ethical standards that advance the good of all are upheld. With increasing reasoning maturity, it would be reasonable to expect that the PI and MN schemas will be employed less in the management of moral issues and greater use of the P schema will be observed [11].

Rest et al. developed the revised iteration of the Defining Issues Test (DIT) [15]. This test aims to evaluate the extent to which a person employs a moral schema to reason through dilemmas. The DIT-2 is an abridged version of the DIT and consists of five dilemmas that requires those undertaking the test to rank and rate statements that relate to each dilemma [16]. The score from the Test provides a value that can be correlated with the varying stages of moral development. The tool has been validated and used in a wide range of educational and non-educational environments [17,18,19]. Scores related to the PI, MN, and P schema can be calculated from the test. A P score greater than or equal to 42 has been suggested as the threshold for a high moral reasoning ability [20]. A new score (N2) has been introduced to the test. It is derived from the degree to which the respondent ranks P items above MN and PI and the difference in ratings of the P score from the PI score [18].

Among the requirements for specialist registration as an orthodontist in Australia, a dentist must have undertaken an approved program of study. Five universities in Australia are approved to provide a 36-month Doctorate of Clinical Dentistry program which satisfies the academic requirements for registration. Orthodontics occupies a unique place in the health provision landscape in that orthodontic intervention is largely elective. This presents ethical challenges to orthodontic treatment providers not experienced in other health disciplines [1]. Although several studies have surveyed opinions from healthcare educators and students regarding professionalism and determined moral reasoning scores from medical, dental, and pharmacy undergraduate students, relevant data from students undertaking specialist dentistry training is lacking [13,16,19,21,22,23,24,25,26,27,28].

The aim of the present investigation was to determine postgraduate orthodontic students’ moral reasoning ability and evaluate their opinions on professionalism and the Dental Board of Australia’s (DBA) tribunal outcomes. The null hypothesis is that the mean P score among the postgraduate students was 42 or greater [20,29].

## 2. Materials and Methods

The cross-sectional investigation was approved by the University of Adelaide Human Research Ethics Committee (approval number H-2024-082). The electronic survey (e-survey) was created on the Qualtrics [Qualtrics Core XM under the Adelaide University academic license] (Seattle, WA, USA) software platform. The design and conduct of the e-survey was in accordance with the recommendations outlined by Eysenbach and Burns et al. [30,31]. The study employed the Strengthening the Reporting of Observational Studies in Epidemiology (STROBE) Statement checklist for cross-sectional studies and was carried out in conjunction with corresponding investigations among U of A undergraduate students [27,28,32].

The e-survey comprised three sections. Section 1 pertained to the non-identifiable demographic characteristics of the respondents. Section 2 contained questions related to respondent knowledge and views of the Code. The section also sought respondent opinions of DBA tribunal outcomes of 10 ‘real life’ cases brought before the DBA from 2013–2023. Only cases brought before the DBA were considered for inclusion in present study as cases that involve the most serious alleged breaches of professional standards, and cases that potentially result in the removal of a dentist’s professional registration, are referred to a tribunal for determination.

Details regarding the cases are available to the public and are accessible through the AHPRA website [33]. On 1 January 2024 all 37 cases with decisions in the time period from 1 January to 31 December 2014 were recorded on a Microsoft (Redmond, WA, USA) Excel spreadsheet as described by Meade and colleagues [27,28]. Each was allocated a number and a random number generator (https://www.calculator.net/random-number-generator.html) (accessed on 1 March 2024) was used to generate 10 cases for the present investigation. Although the cases could be categorised according to more than one theme, they were determined to relate to the broad themes of consent (Cases 1 and 5), protection of the title (Cases 2 and 9), financial fraud (Cases 3 and 8), and (potential) negligence (Cases 4, 6, 7, and 10). The respondents were asked to answer whether they believed that the outcome was very harsh, harsh, fair, lenient, or very lenient.

The survey questions in Sections 1 and 2 were adapted, with permission, from a similar study among undergraduate pharmacy students in the United Kingdom and involved pre-piloting among dental clinician colleagues [16].

Sections 1 and 2 were further piloted among two postgraduate orthodontic students and an orthodontist who had just completed postgraduate orthodontic specialist training. The students and the orthodontist provided feedback related to the pertinence and clarity of questions and how long it took to complete the survey. The mean time for survey completion was less than 30 min.

Permission was provided by the University of Alabama to use the DIT-2 [18]. Section 3 contained the questions related to, and comments following, the five DIT-2 dilemmas:

1. A dad considering thieving food for his hungry dependents.
2. A journalist contemplating if he should publish a damaging article concerning a politician.
3. A school board chairperson deciding if he should proceed with holding a controversial meeting open to the public.
4. A medic faced with a request by a patient, who is suffering, to provide him with an overdose of analgesia.
5. College pupils protesting a foreign policy.

Respondents were required to rank (from 1 to 4) and rate (using a five-point scale) the significance of the questions and comments. Section 3 also required respondents to self-report their political orientation according to one of the following descriptors: ‘very conservative’, ‘conservative’, ‘neither conservative nor liberal’, ‘liberal’, or ‘very liberal’.

As the DIT-2 tool is a copyrighted and validated instrument, it underwent minimal change during the pre-piloting and piloting stages.

The survey was disseminated to the 46 postgraduate orthodontic students undertaking their orthodontic studies at the Universities of Adelaide, Western Australia, Sydney, Queensland, and Melbourne via the academic directors of the orthodontic programs in each university. A link to the e-survey with a cover letter and a participation information sheet was sent to the eligible populations in the week beginning 28 October 2024. A reminder was sent by the directors in the week beginning 18 November. The survey was closed on 30 November 2024. All respondents provided informed consent to participate in the survey and those who completed the surveys were provided with the opportunity to enter a prize draw for $250. All responses to the survey were anonymous and responses to the e-survey could not be linked to prize draw entrants.

The responses related to the DIT-2 were entered into a pre-formatted Microsoft Excel spreadsheet and electronically transmitted to the University of Alabama for scoring. The responses from Sections 1 and 2 were also entered into a Microsoft Excel spreadsheet where coding was undertaken to ensure that the results from the University of Alabama could be matched with the correct respondents from the first two Sections.

Statistical analyses were carried out using SPSS (version 27) (IBM, Armonk, NY, USA). Calculation via Yamane’s formula (sometimes called Slovin’s formula) indicated that a response of 85.8% (n > 39) would provide a margin of error of 5% [34]. Descriptive statistics were presented in text and tabular formats. Means, standard deviations (SD) and 95% confidence intervals (CIs) as well as medians and inter-quartile range (IQR) were calculated. The Shapiro–Wilks test was used to determine the normality of data distribution. A p score greater than 50 is considered to be moral reasoning at a high level, with moral dilemmas being addressed in a manner analogous to that adopted by a moral philosopher [35]. However, a p score greater than or equal to 42 has been considered a threshold for high moral reasoning ability in the present study [20].

The Mann–Whitney U test was used to test for significance between medians, and the Spearman’s rank correlation coefficient was used to determine whether there was a correlation between the opinions on professionalism and DBA tribunal outcomes. The interpretations of the absolute values of the correlation coefficients were:

- 0.0 to 0.09: negligible correlation
- 0.10 to 0.39: weak correlation
- 0.40 to 0.69: moderate correlation
- 0.70 to 0.89: strong correlation and
- 0.90 to 1.0 very strong correlation [36].

Statistical significance was set at *p* < 0.05.

## 3. Results

A total of 37 postgraduate students responded to the survey. This corresponded to a response rate of 80.4%. According to Yamane’s formula, this provided a margin of error of 6%. The Shapiro–Wilks test indicated that data distribution was non-parametric. The majority of respondents were females (n = 20; 54.1%) with 16 males (43.2%) and one (2.7%) ‘prefer not to say’ making up the remainder. The median age (IQR) was 30.0 (29.0, 33.0) years. There was no difference (*p* = 0.71) between the median (IQR) ages of female [30.0; 30.0, 33.0] and male [31.0; 28.5, 35.0] respondents.

All respondents (n = 37; 100%) answered that they were aware of the DBA Code of Conduct. Table 1 outlines the responses made by the students to the ‘Professionalism’ statements.

Table 2 provides an overview of the details and responses to the 10 DBA outcomes.

The scores from the DIT-2 questionnaires of two students were not calculated by the University of Alabama as insufficient or no responses were provided. Table 3 outlines the number of students who self-reported their political orientation.

Table 4 provides an overview of the DIT-2 scores. Males recorded significantly higher scores for P scores (males—median (IQR) [31.0, (19.5, 46.0); females—median (IQR) [28.0, (20.0, 32.0) *p* < 0.001] and N2 scores (males—median (IQR) [28.3, (19.8, 43.9); females—median (IQR) [24.7, (14.12, 34.74) *p* < 0.001].

There was a weak correlation between N2 scores and self-reported political orientation (r = 0.29; 95% CI: −0.30 to 0.72) and between P scores and self-reported political orientation (r = 0.35; 95%CI: 0.01 to 0.62). There was a negligible correlation between age and N2 scores (r = 0.02; 95% CI: −0.33 to 0.37) and a weak correlation between age and P scores (r = 0.28; 95%CI: −0.08 to 0.57).

Table 5 shows that there was a medium correlation between the P score and Professionalism statement 8, which related to the students’ responses about fully valuing their experiences at university and compliance with the Code. In addition, a weak correlation was noted between the P score and DBA outcome, which concerned dishonest evidence given to a court.

## 4. Discussion

The aim of the present investigation was to determine Australian postgraduate orthodontic students’ moral reasoning ability and evaluate their opinions on professionalism and the outcomes from the DBA’s tribunal outcomes. The findings indicated that the moral reasoning scores of the postgraduates were lower than the minimum suggested for high moral reasoning and that respondent opinions regarding DBA outcomes were that they were fair or tended towards being lenient. The data from this investigation provide baseline information in relation to moral reasoning and ethics education for postgraduate orthodontic students and insight into registrants’ opinions about the outcomes from the profession’s health professional regulator. The null hypothesis was not accepted.

Relevant data related to students undertaking specialist dentistry training and from research undertaken in Australia are limited. However, comparison with investigations undertaken in other healthcare disciplines and in other countries can provide some insight. Consistent with most moral-reasoning studies, we report the mean P score; in our sample, it was 31.1 and was less than the 42 proposed by Rest for superior moral reasoning and the score considered as the minimal acceptable in the current study [29]. This compared with findings of 32.4 to 41.6 among dentistry students in the US [13], Australia [28], and Brazil [22], and dental hygiene students in Korea [37]. However, the scores were less than the 40 observed among new paediatric resident physicians in a hospital facility in Mexico [38], 51.9 in a study regarding student applications for a position on an orthopaedic surgery resident program in the US [10], and 47 among nursing students in Finland [39]. The relatively poor scores in the present study aligned with scores from different moral reasoning scoring instruments employed among dental students in Saudi Arabia [40] and Iran [41]. However, it contrasted with the findings from an investigation [42] among general and post-graduate dental students in the Southeast of Iran where the authors considered the responses related to moral reasoning to the ‘Moral Skills and Inventory’ questionnaire [43] to be acceptable.

Although the literature has shown a tendency towards slightly higher moral reasoning scores among females, the findings are often inconsistent [18]. No differences between males and females, for example, have been found in corresponding investigations among individuals undertaking education in dentistry [13,28,40], pharmacy [16], paediatric physician training [38], and nursing [39]. Why males recorded slightly higher moral reasoning scores in the study presented here warrants further investigation. However, variation in scores may (at least in part) be due to the complex interplay of age, level of education, cultural factors and the type of healthcare discipline undertaken.

Relevant data regarding moral reasoning among clinicians in dental practice are limited [28,44] and are lacking in specialist orthodontic practice. Comparison with pharmacy practice, however, may provide some limited value, with the results in the present study corresponding to scores of 25 to 33 among pharmacy students in the US [45], England [46], and Northern Ireland [16]. A proposed explanation for the relatively low scores recorded in these three investigations may relate to the circumstances in which the practice of pharmacy is generally conducted—namely, business-focused and transactional [47,48], in an environment independent from other healthcare providers, rather than exclusively concentrated on healthcare provision [16,49]. This may help explain the P score in the present study as dental and orthodontic services in Australia, including those provided in undergraduate and postgraduate dentistry programs, generally require patients to contribute to the costs of their treatment.

Moreover, the nature of the environment in which students in other healthcare disciplines conduct clinical activity may have facilitated more instant encounters with matters that necessitated in-depth ethical consideration where the development of more sophisticated moral reasoning skills occurs [10,16,46]. Postgraduate orthodontic students, for example, are not confronted with the ‘life or death’ instant decision-making seen in other health disciplines. A study [39] among nursing students in Finland indicated that those who had had to address ethical dilemmas during training had higher moral judgment scores than those who did not. Furthermore, the requirement to adhere to protocols and regulations of controlled clinical and educational domains may have inhibited the development of, or resulted in the regress of, moral reasoning skills among the postgraduate orthodontic students [46,50,51].

In addition, the findings from a study by Self et al. [50] indicated that the curriculum and teaching context may constrain rather than develop moral reasoning because the learning environment encourages ‘convergent thinking’ [52]. Convergent thinking focuses on seeking the right answer, instead of probing and questioning. Self and colleagues determined that conservation of the rules of the system was essential. This corresponded to a ‘conventional level’ moral ethos in a cognitive moral theory context and may also explain the lower score observed in this and other studies. This suggests that Kohlberg’s concept of moral reasoning undervalued the role of relationships [46]. However, a ‘rules-based’ system may have helped ensure the high rates of agreement of the respondents’ understanding of professionalism and the need to comply with the Code of Conduct in the present study.

Respondents were broadly in agreement with the Professionalism statements and this aligned with the responses to similar statements from undergraduate pharmacy students in Northern Ireland [16] and undergraduate dentistry students in Australia [27]. Almost one in six (15.4%), however, believed that adherence to the Code meant that they could not fully value the student experience while at university. This may have reflected the conflict that some registered healthcare professionals experience in applying the professionalism standards set by their regulatory authority in their private lives [4,7,53,54,55,56].

Most respondents believed that most DBA tribunal outcomes were fair or tended towards being lenient. This was in alignment with the findings from the study by Hanna and colleagues [16], where a similarly cautious approach was observed in respondent decision-making. The students opined that the outcomes of the two cases relating to financial fraud (3 and 8) were harsh. As the outcome did not relate to a situation involving direct patient harm, the students may have considered that the outcome was excessively punitive. This contrasted with those cases where there was a more obvious risk of patient harm or actual harm observed. For example, over half of the respondents indicated that the outcome in Tribunal 5, where a dentist was found to have failed to obtain informed consent from the guardians or responsible persons for five patients with intellectual disabilities, was very lenient or lenient. This suggested the importance students placed on valid consent in the provision of care [57]. Additionally, almost 70% of the respondents believed a fine of $11,620 was either lenient or very lenient for a non-registered individual who indicated that he was a dentist and performed several restricted acts. This likely indicated a concern for patient welfare. It may also have been an acknowledgement that ‘dentist’ is a protected title under Australian law which provides reassurance to the public that users of the title have attained the appropriate qualifications and are satisfying the requirements for registration with the AHPRA [58]. Furthermore it may have illustrated a ‘personal freedom of action’ dimension, independent of theoretical moral reasoning and adherence to a code of professional standards, whereby the students felt freer to express an opinion that ‘contravened’ the judgement of a regulatory authority [47].

The medium correlation between higher P score and Professionalism statement 8, regarding the students’ responses about fully valuing their experiences at university and compliance with the Code, indicated a potential tension between ethics and professional regulation. Further investigation is required to determine whether the adherence to standards of professionalism related to students’ broader life experiences is a greater source of conflict and questioning in those with higher moral reasoning scores.

The findings of the present study indicated that improvement in the moral reasoning skills of postgraduate orthodontic students in Australia is required. Currently, there is little formal training in professional ethics in the five postgraduate orthodontic training programs in Australia. The incorporation of relevant formal teaching may contribute to amelioration in this regard. Latif [59] showed that the introduction of ethical dilemma case discussions improved the moral reasoning skills of pharmacy undergraduates and concluded that these skills are teachable. Self and colleagues demonstrated that medical students undergoing 20 or more hours of case study discussions in small groups related to medical ethics increased the students’ moral reasoning scores. More broadly, further education in professional ethics may help students be more sensitive to the moral aspects of professional practice, enable the students to appreciate and accept ambiguity, emphasise the moral responsibilities of being a specialist orthodontist, and evoke an impression of moral obligation [60,61]. The outcome of education in moral reasoning may be an increase in curiosity, questioning, and exploration of ethically challenging issues that the students face as their moral reasoning skills develop. It may help provide the students with the tools to negotiate those issues that may potentially conflict with the regulatory agency’s Code of Conduct.

The shortcomings of the present survey must be acknowledged. Although the response rate was very high, it may have been higher if a guaranteed smaller monetary incentive had been offered to all respondents rather than entry into a prize draw [62]. Furthermore, the findings from a 2017 study [63] indicated that students were more likely to respond to if they found the topic to be of interest and that their responses would make a difference, rather than the possibility of winning a prize. In addition, social desirability is a risk with all surveys. However, respondent anonymity and the nature of the DIT-2, whereby respondents are unlikely to have the ability to ‘fake’ higher moral reasoning scores, minimises the risk [64]. A further limitation is the lack of evaluation of the partially completed questionnaires. Data in this regard are not provided by the University of Alabama’s statistical analysis and, consequently, may have impacted the present study’s findings. As the population surveyed was relatively small and restricted to a specific postgraduate dental academic program in Australia, the findings have limited generalizability. Statistical analyses were largely exploratory, which means caution is required in interpreting the results. Future research should investigate whether the findings are likely to be replicated in the postgraduate programs of other dental specialties in Australia and other countries with similar regulatory frameworks. Moreover, it is important to note that the findings from studies such as this may be liable to false negatives and false positives [10]. Orthodontists with high P scores do not necessarily have perfect moral reasoning, nor do those with low P scores automatically demonstrate a shortcoming in moral reasoning that adversely affects patient care. Moral reasoning is just one of the four psychological capacities that underpin the theory of moral behaviour developed by Rest and colleagues from Kohlberg’s stages of moral development [11,12]. Future investigations should consider the role of moral sensitivity, moral courage, and moral integrity in ethical behaviour in the orthodontic context [43]. Further research should also incorporate moral reasoning abilities, opinions on DBA tribunal outcomes and ethical decision-making of qualified clinicians in specialist orthodontic practice is required.

However, the findings of the present study provide insight into the moral reasoning ability and the opinions of postgraduate orthodontic students regarding DBA tribunal outcomes. They also provide baseline data for future investigations related to the moral reasoning and ethical behaviour of students in postgraduate orthodontic and other postgraduate dental specialties, as well as clinicians in dental and dental specialty practice.

## 5. Conclusions

• The moral reasoning scores were lower than what may be expected from individuals in the provision of healthcare.
• Respondents were broadly supportive of the statements related to professionalism, although 15.4% believed that adherence to the Code meant that they could not fully value the student experience while at university.
• Respondent opinions regarding DBA outcomes tended towards fair or lenient, although they felt that outcomes related to informed consent and the protected title were very lenient, while one outcome regarding dishonest evidence by a dentist in court related to an employment dispute was considered harsh or very harsh by 53%.
• The introduction of formal training in moral reasoning may develop postgraduate moral reasoning skills and DIT-2 scores.

## Figures and Tables

**Table 1:** Responses to ‘Professionalism’ statements (n = 37).

Statement [16]	Agreement/Disagreement				
	Strongly Agree	Agree	Neither Agree nor Disagree	Disagree	Strongly Disagree
	n (%)	n (%)	n (%)	n (%)	n (%)
(1) ‘I have a complete understanding of what is meant by the term “professionalism”’.	18 (48.6)	19 (51.4)	0	0	0
(2) ‘During the DCD degree, I have been made completely aware of the professional behaviour that is expected of me.’	22 (59.5)	11 (29.7)	4 (10.8)	0	0
(3) ‘I fully understand what my Dental School classifies as unacceptable professional behaviour.	22 (59.5)	14 (37.8)	1 (2.7)	0	0
(4) ‘I understand why complying with the standards set out in the Code is a necessary part of the DCD program.’	28 (75.7)	7 (18.9)	2 (5.4)	0	0
(5) ‘It is reasonable that the Code of Conduct applies to me at all times i.e. whether I am studying within university or out socializing with friends.’	20 (54.1)	13 (35.1)	4 (10.8)	0	0
(6) ‘It is fair that the DBA is informed about breaches to the Code.’	24 (18.9)	12 (32.4)	1 (2.7)	0	0
(7) ‘The Code is a major influence on my behaviour when I am out socializing with friends.’	11 (29.7)	9 (24.3)	8 (21.6)	6 (16.2)	3 (8.1)
(8) ‘Being bound by the Code means that I cannot fully value the student experience whilst at university.’	4 (10.8)	2 (5.4)	12 (32.4)	9 (24.3)	10 (27.0)

KEY. n: number; DCD: Doctorate of Clinical Dentistry (Orthodontics); Code: Code of Conduct; DBA: Dental Board of Australia.

**Table 2:** Responses to DBA tribunal outcomes (n = 37).

	Response				
DBA Tribunal Details and Outcomes [28]	Very Lenient	Lenient	Fair	Harsh	Very Harsh
	n (%)	n (%)	n (%)	n (%)	n (%)
‘Dr K administered multiple anaesthetic injections causing “a surge of intense pain” to Patient P. After being advised to stop the treatment, he did not, despite assurances that he would stop if she was in pain. He then continued onto a second injection, resulting in excruciating pain to P. Dr K continued the dental procedure after P had withdrawn consent, failed to apply adequate or effective local anaesthesia for P’s tooth to be extracted safely; failed to extract the tooth in line with contemporary extraction practices; and failed to act courteously, respectfully and compassionately with Ms P.’ *Outcome: Reprimanded and disqualified for nine months.*	3 (8.1)	8 (21.6)	24 (18.9)	2 (5.4)	0
2. ‘A dentist whose registration with the Dental Board of Australia (AHPRA) lapsed in 2018, pleaded guilty in the Magistrates Court of Victoria to three charges of holding himself out as a dentist when he was not registered and eight charges of performing restricted dental acts, including orthodontic treatment, on several patients.’ *Outcome: A fine of $11,620.*	8 (21.6)	18 (48.6)	11 (29.7)	0	0
3. ‘A dentist was paid approximately $169,480 by a health insurer after making 1915 claims for dental procedures between June and December 2011 that she had not provided. The fraudulent conduct involved the dentist maintaining false, misleading and inaccurate medical records, recording procedures or tests that had not been performed, and obtaining and signing consent forms for procedures and tests that did not occur.’ *Outcome: Reprimanded and disqualified from applying for registration as a registered health practitioner for five years.*	1 (2.7)	2 (5.4)	27 (73.0)	7 (18.9)	0
4. ‘A dentist attended a nightclub with a patient and communicated about matters that were unrelated to the practitioner/patient relationship. The dentist also admitted that she had induced a state of conscious sedation in the patient by prescribing Midazolam and Temazepam when she did not hold the relevant endorsement for conscious sedation from the Dental Board of Australia (AHPRA), and when she did not have the relevant education or training to induce a state of conscious sedation. The dentist further admitted that she had failed to maintain adequate clinical records in relation to treatment she had provided to the patient.’ *Outcome: Reprimanded and a requirement to be mentored by another health practitioner with a review at 6 months. Further education on clinical record keeping and scope of practice.*	2 (5.4)	16 (43.2)	16 (43.2)	3 (8.1)	0
5. ‘A dentist was found to have failed to obtain informed consent from the guardians or responsible persons for five patients with intellectual disabilities who could not effectively communicate and did not have capacity to provide consent or make decisions about their dental treatment. The dentist also failed to provide adequate follow-up care, inappropriately charged for the examinations and treatment, and failed to maintain adequate clinical records.’ *Outcome: Reprimanded with registration suspended for three months and education conditions relating to informed consent.*	5 (13.5)	15 (40.5)	14 (37.8)	3 (8.1)	0
6. ‘A dentist self-administered schedule eight medicines (fentanyl and pethidine) that he had obtained in his professional capacity as a dentist and practice principal. He was found to have had demonstrated insight into the serious nature of his actions by admitting to them, expressing remorse, and cooperating with the AHPRA and the Office of the Health Ombudsman (co-regulator of registered dental practitioners based in Queensland).’ *Outcome: Reprimand with a recommendation that the reprimand is removed after 12 months*	7 (18.9)	2 (5.4)	28 (75.7)	0	0
7. ‘A dentist failed to adequately care and treat six of his patients at his practice. This included failing to make appropriate arrangements for timely treatment resulting in significant delay in receiving a permanent crown, failing to pay for and obtain permanent crowns that had been pre-paid for by two patients, failing to provide an adequate treatment plan, failing to provide adequate or timely receipts, and failing to produce patient records when requested to do so by the APHRA.” *Outcome: Reprimand, removal of name from the register of practitioners and banned from reapplying for registration for two years*.	1 (2.7)	20 (54.1)	14 (37.8)	1 (2.7)	1 (2.7)
8. ‘Dr H gave dishonest evidence to a local court in proceedings he had brought against his former employer for unpaid payments under an employment contract for work in Darwin. The dishonest evidence related to the Dr H’s claim to his former employer that he could no longer work on Saturdays for personal reasons, when in fact he was moonlighting as a dentist in Singapore when he should have been practising in Darwin. Dr H’s case was that he did not work as a dentist in Singapore until after the contract was terminated. The tribunal found that “the knowingly false evidence was intended to, and probably did, assist Dr H in achieving success in that litigation”.’ *Outcome: Disqualified from applying for registration for five years, banned from working in certain health services and using specified titles and ordered to pay $100,000 in costs.*	0	0	17 (45.9)	18 (48.6)	2 (5.4)
9. ‘An individual who never held registration as a dentist in Australia was found to be holding himself out as a dentist and carrying out restricted dental acts. The acts occurred in a makeshift dental clinic at his home in contravention of the Health Practitioner Regulation National Law.’ *Outcome: Suspended from register for 3 months*.	25 (67.6)	12 (32.4)	0	0	0
10. ‘A dentist has been reprimanded after he was found to have performed a procedure for which he did not have sufficient expertise or experience. It was determined that he performed gingival laser depigmentation incorrectly, failed to initially treat a small patch of gingival tissue, and performed the procedure when he did not have sufficient expertise or experience of using a diode laser in gingival laser depigmentation and demonstrated inadequate clinical record-keeping.’ *Outcome: Reprimanded with conditions on his registration that he must complete an education program, provide a detailed reflective practice report to the AHPRA and ordered to pay $3500 towards the AHPRA’s costs.*	3 (8.1)	12 (32.4)	22 (59.5)	0	0

**Table 3:** Self-reported description of political orientation (N = 35).

Political Orientation	N	%
Very liberal	3	8.6
Somewhat liberal	10	28.6
Neither conservative nor liberal	15	42.9
Somewhat conservative	5	14.3
Very conservative	2	5.7

Key. N: number; %: percentage.

**Table 4:** Overview of DIT-2 scores (N = 35).

	Mean	Std. Error of Mean	Median	SD	Variance	Range	Min	Max
Political orientation	2.80	0.17	3.00	0.99	988	4.00	1.00	5.00
P score	31.14	2.24	30.00	13.25	175.60	46.00	10.00	56.00
PI (Stage 2/3)	30.00	2.06	32.00	12.20	148.94	48.00	4.00	54.00
MN (Stage 4)	29.89	1.74	30.00	10.30	106.10	48.00	52.00	6.45
N2 score	28.026	2.247	27.66	13.29	176.67	50.61	6.45	57.07

KEY: DIT: defining issues test; N: number; Std: standard; SD: standard deviation; Min: minimum; Max: maximum; P: post-conventional score; PI: personal interests score; MN: maintaining norms.

**Table 5:** Correlation of student responses to Professionalism statements and DBA outcomes to DIT-2 P and N2 scores.

Statement/Outcome Responses	Moral Reasoning Postconventional Schema	
	Spearman Correlation Coefficient	
	(‘P Score’)	N2 SCORE
	R (95% CI)	R (95% CI)
Professionalism—statement 1	0.07 (−0.28 to 0.41)	0.12 (−0.24 to 0.44)
Professionalism—statement 2	0.19 (−0.17 to 0.50)	0.07 (−0.28 to 0.41)
Professionalism—statement 3	0.10 (−0.26 to 0.43)	<0.01 (−0.3460 to 0.3487)
Professionalism—statement 4	0.37 (0.032 to 0.64)	0.26 (−0.092 to 0.56)
Professionalism—statement 5	<−0.01 (0.35 to 0.34)	−0.05 (−0.39 to 0.31)
Professionalism—statement 6	0.18 (−0.18 to 0.49)	0.05 (−0.30 to 0.39)
Professionalism—statement 7	−0.08 (−0.42 to 0.28)	−0.27 (−0.56 to 0.086)
Professionalism—statement 8	−0.40 (−0.66 to −0.062)	−0.22 (−0.52 to 0.14)
Outcome 1	0.17 (−0.39 to 0.30)	0.036 (−0.32 to 0.38)
Outcome 2	−0.05 (−0.39 to 0.30)	−0.18 (−0.50 to 0.18)
Outcome 3	−0.27 (−0.57 to 0.083)	−0.14 (−0.46 to 0.22)
Outcome 4	0.31 (−0.28 to 0.73)	−0.05 (−0.39 to 0.30)
Outcome 5	−0.07 (−0.41 to 0.28)	−0.26 (−0.56 to 0.092)
Outcome 6	−0.16 (−0.49 to 0.19)	−0.33 (−0.61 to 0.02)
Outcome 7	0.13 (−0.22 to 0.46)	0.01 (−0.34 to 0.36)
Outcome 8	0.10 (−0.25 to 0.44)	0.17 (−0.19 to 0.49)
Outcome 9	0 (−0.35 to 0.35)	−0.07 (−0.41 to 0.29)
Outcome 10	<0.01(−0.35 to 0.34)	−0.09 (−0.43 to 0.26)

Key. DBA: Dental Board of Australia. DIT: defining issues test. P: post-conventional score. R: rho. CI: confidence interval.

## Data Availability

The original contributions presented in this study are included in the article. Further inquiries can be directed to the corresponding author.

## Abbreviations

U of A	University of Adelaide
DIT	Defining Issues Test
DBA	Dental Board of Australia
DCD	Doctor of Clinical Dentistry
CI	Confidence Interval
MN	Maintaining Norms
STROBE	Strengthening the Reporting of Observational Studies in Epidemiology
IQR	Interquartile
